# Why Do Public Safety Personnel Seek Tailored Internet-Delivered Cognitive Behavioural Therapy? An Observational Study of Treatment-Seekers

**DOI:** 10.3390/ijerph182211972

**Published:** 2021-11-15

**Authors:** Hugh C. McCall, Caeleigh A. Landry, Adeyemi Ogunade, R. Nicholas Carleton, Heather D. Hadjistavropoulos

**Affiliations:** 1 Department of Psychology, University of Regina, 3737 Wascana Pkwy, Regina, SK S4S 0A2, Canada; Hugh.McCall@uregina.ca (H.C.M.); Caeleigh.Landry@uregina.ca (C.A.L.); Nick.Carleton@uregina.ca (R.N.C.); 2 PSPNET, University of Regina, 2 Research Drive, Regina, SK S4T 2P7, Canada; Adeyemi.Ogunade@uregina.ca

**Keywords:** internet, cognitive behavioural therapy, anxiety, depression, eHealth, public safety personnel

## Abstract

First responders and other public safety personnel (PSP) experience elevated rates of mental disorders and face unique barriers to care. Internet-delivered cognitive behavioural therapy (ICBT) is an effective and accessible treatment that has demonstrated good treatment outcomes when tailored specifically for PSP. However, little is known about how PSP come to seek ICBT. A deeper understanding of why PSP seek ICBT can inform efforts to tailor and disseminate ICBT and other treatments to PSP. The present study was designed to (1) explore the demographic and clinical characteristics, motivations, and past treatments of PSP seeking ICBT, (2) learn how PSP first learned about ICBT, and (3) understand how PSP perceive ICBT. To address these objectives, we examined responses to online screening questionnaires among PSP (*N* = 259) who signed up for an ICBT program tailored for PSP. The results indicate that most of our sample experienced clinically significant symptoms of multiple mental disorders, had received prior mental disorder diagnoses and treatments, heard about ICBT from a work-related source, reported positive perceptions of ICBT, and sought ICBT to learn skills to manage their own symptoms of mental disorders. The insights gleaned through this study have important implications for ICBT researchers and others involved in the development, delivery, evaluation, and funding of mental healthcare services for PSP.

## 1. Introduction

Public safety personnel (PSP) include border services officers, public safety communications officials, correctional workers, dispatch/communication workers, career and volunteer firefighters, Indigenous emergency managers, operational intelligence personnel, paramedics, police (municipal, provincial, and federal), search and rescue personnel, and others [1]. Through their work, PSP are frequently exposed to potentially psychologically traumatic events, and such exposures predict symptoms of various mental disorders [2,3]. A large-scale national survey showed that 44.5% of Canadian PSP reported clinically significant symptoms of at least one mental disorder [4]. There is also evidence of high rates of mental health problems among PSP in other countries, including Australia [5], Brazil [6], the Netherlands [7,8], Taiwan [9], and the United States [10,11]. PSP report unique barriers to accessing mental healthcare, such as concerns about stigma and workplace repercussions for seeking help and distrust of, or discomfort with, mental healthcare providers [12,13,14].

Despite recent efforts at reducing mental health stigma among PSP groups (e.g., the Road to Mental Readiness program [15]), stigma remains an important barrier to treatment-seeking among PSP [16]. The most prominent concerns related to stigma include concerns regarding the confidentiality of sessions and fear that seeking services would have a negative impact on their careers [16], as people may believe seeking care makes them discreditable by their peers (e.g., weak, lazy, deceitful, ill-suited for their job) [17]. Stigma has further been related to increased alcohol use in PSP, which can cause detrimental impacts on their careers [16]. As such, stigma may prevent PSP from seeking help or time off work [14].

There is growing interest in using internet-delivered cognitive behavioural therapy (ICBT) to treat mental health problems among PSP. Hundreds of trials over the past two decades have demonstrated that ICBT is effective for treating symptoms of several disorders, including anxiety, mood, and trauma-related disorders [18,19,20]. ICBT is typically accessed through a web browser, using similar security features as online banking to ensure protection of clients’ sensitive information [20]. It can be offered with therapist guidance, generally by email or phone, or in an unguided, purely self-help format. Meta-analyses have shown that ICBT is similarly efficacious to face-to-face cognitive behavioural therapy [21,22]. ICBT is private, can be accessed at any time and location, and is cost-effective, facilitating access for people who face barriers to other forms of care (e.g., stigma, inconvenience, affordability) [23,24]. There are now successful ICBT clinics in Australia, Canada, Denmark, Norway, and Sweden, as well as thousands of smartphone apps designed to treat mental health problems, many of which use an ICBT approach [20,25]. However, many implementation efforts have been hindered by issues such as high attrition rates, lack of consensus concerning the necessary standards of evidence to support wider implementation, and problems with study designs [26]. PSP have reported favorable attitudes towards ICBT, describing the privacy and convenience it affords as particular advantages [12,27]. Our research unit, PSPNET, has developed a transdiagnostic ICBT program tailored specifically for Canadian PSP, in keeping with recommendations to tailor digital mental health interventions towards specific user groups (e.g., [28,29]). PSPNET’s ICBT has demonstrated promising initial outcomes for treating symptoms of several mental disorders among PSP [30]. At the time this article was written, at least two other Canadian research groups were also developing ICBT interventions for PSP [31,32].

Currently, very little is known about how PSP come to seek ICBT; however, establishing a clearer understanding of PSP’s ICBT-seeking experiences can help inform efforts at tailoring and disseminating ICBT to PSP. For instance, better understanding the challenges PSP experience can facilitate more effective tailoring of treatment content, while better understanding PSP’s reasons for seeking ICBT can guide efforts to educate PSP about it. Likewise, identifying the underrepresentation of any demographic or occupational PSP groups among ICBT seekers can reveal a need for additional outreach efforts, and evaluating perceptions of ICBT among ICBT seekers is important because treatment perceptions have been shown to predict treatment outcomes [33,34,35].

By examining the responses of prospective PSPNET clients to eligibility screening questionnaires, the current study was designed to address several questions related to ICBT-seeking among PSP: (a) What demographic and occupational groups do prospective PSPNET clients belong to? (b) How did prospective clients first hear about PSPNET? (c) How do prospective clients perceive ICBT? (d) What motivations do prospective clients report for seeking ICBT? (e) What are the current clinical characteristics of prospective PSPNET clients? (f) What past and current mental health treatments do PSP report having tried? In an effort to answer each of these questions as comprehensively as possible, we employed a mixed-methods approach. Answering these questions will help determine how ICBT might better address the alarming rates of mental health problems among PSP in Canada and other countries.

## 2. Materials and Methods

### 2.1. Setting

PSPNET began offering services across Saskatchewan in December 2019 and across Quebec in September 2020. When the current study was conducted, there were no other ICBT services tailored for PSP in Canada; however, ICBT was freely available to the general population in Saskatchewan through the Online Therapy Unit [36]. There were also several paid and unpaid ICBT services available to the general population across Canada [37].

### 2.2. Participants and Procedures

Participants in the present study were all prospective PSPNET clients enrolled between December 2019 and March 2021. Before enrolling in one of PSPNET’s ICBT courses, PSP are required to complete a series of questionnaires online for eligibility screening purposes (details below). All of the current results are based on data obtained from the eligibility screening questionnaires. The procedures through which PSP enrol in and complete PSPNET courses are described elsewhere [30].

### 2.3. Measures

Symptoms of major depressive disorder (MDD) were measured using the Patient Health Questionnaire-9 (PHQ-9) [38]. Symptoms of generalized anxiety disorder (GAD) were measured using the Generalized Anxiety Disorder-7 (GAD-7) [39]. Other symptom measures included the Posttraumatic Stress Disorder Checklist for the DSM-5 (PCL-5), which measured symptoms of PTSD [40]; the Panic Disorders Severity Scale—Revised Self-Report (PDSS-SR), which measured symptoms of panic disorder [41]; the Social Phobia Scale (SPS-6) and the Social Interaction Anxiety Scale (SIAS-6), which are used together to measure symptoms of social anxiety disorder [42]; the French version of the Social Interaction Phobia Scale (SIPS), which was used to measure symptoms of social anxiety disorder among francophone PSP, because we were unable to find a French version of the SIAS-6 and SPS-6 [43,44,45]; and the Dimensions of Anger Reaction Scale-5 (DAR-5), used to assess anger [46]. Alcohol and drug use were measured using the Alcohol Use Disorders Identification Test (AUDIT) [47] and the Drug Use Disorders Identification Test (DUDIT) [48], respectively. Current functional impairment due to mental health problems was measured using the Sheehan Disability Scale (SDS) [49]. The Credibility and Expectancy Questionnaire (CEQ) was used alongside bespoke questions inquiring about prospective clients’ perceptions and expectancies of ICBT [50]. The Canadian Adapted Treatment Inventory of Costs in Patients with psychiatric disorders (TiC-P) was used to measure health services use and productivity loss due to health problems [51]. Participants were asked how they heard of ICBT and why they were interested in ICBT using a bespoke questionnaire. Characteristics of these questionnaires are described in Table 1 below.

### 2.4. Data Analysis

We analyzed data using a mixed-methods approach. We used a quantitative approach to address all research questions, and we used a qualitative approach to address our research question concerning PSP’s motivations for seeking ICBT. We excluded prospective clients from our analyses who did not complete the three primary symptom measures (i.e., PHQ-9, GAD-7, PCL-5) or did not report living in Saskatchewan or Quebec at the time of completing the eligibility screening questionnaires. For clients who enrolled in the eligibility screening multiple times, we used only data from their first enrolment. We compared characteristics of PSP who ultimately enrolled in an ICBT course with PSP who did not, using a series of chi-square tests for categorical variables and *t*-tests for continuous variables. Specifically, we compared enrollers and nonenrollers on the following variables: scores on all symptom measures (i.e., PHQ-9, GAD-7, PCL-5, PDSS-SR, SIAS-6/SPS-6, French SIPS, DAR-5, AUDIT, and DUDIT); diagnostic status on all symptom measures; number of symptom measures for which a client’s score was in the clinical range; SDS scores; CEQ scores; past-year and lifetime use of mental healthcare programs, services, and providers or hospitalizations for mental health concerns; current support from a mental healthcare provider; currently being on a waitlist to see a mental healthcare provider; current medication use for mental health concerns; presence of physical health issues; number of reasons each PSP reported being interested in seeking ICBT; and demographic variables (i.e., whether each PSP has children, community size, level of education, income, relationship status, ethnicity, employment status, and gender). We also compared men and women on all of the demographic variables except for gender. Due to the number of comparisons, alphas were set to 0.01 to control for spurious inflation of Type 1 error. There were minimal differences between PSP who ultimately enrolled in an ICBT course and those who did not, so we present data for the combined sample throughout this paper. Ultimately, we identified only one statistically significant difference between these groups and one statistically significant difference between men and women, both of which we report in the Results section below.

We conducted a thematic analysis of participants’ textual responses to the question “What is your primary motivation for seeking Internet-delivered cognitive behaviour therapy at this time?”. We chose to use thematic analysis as a theoretically flexible qualitative method that can be applied within a positivist/quantitative paradigm or a qualitative paradigm without sacrificing methodological rigor [52]. To ensure coding reliability, authors A.O. and C.L. both conducted a thematic analysis [53,54]. We used the software NVivo 12 [55]. A.O. and C.L. independently established themes by identifying units of meaning expressed by two or more participants and then met to reconcile any differences in the codebook. First, authors A.O and C.L. engaged in data familiarization, whereby they read and reread participant responses and wrote familiarization memos to understand and become immersed in the data. Second, authors A.O and C.L carried out initial coding independently and developed codebooks to reflect their interpretations of the data. Third, A.O. and C.L. independently developed themes that represented initial codes or units of meaning expressed by two or more participants. Fourth, A.O and C.L named, defined, and organized these themes independently into codebooks. Subsequently, A.O and C.L met over multiple sessions to reconcile differences in their individual codebooks and to ensure intercoder reliability in the final codebook. After finalizing the codebook, A.O. and C.L. reviewed—and, in some cases, reanalyzed—the data to help ensure the data were coded consistently.

## 3. Results

### 3.1. What Demographic and Occupational Groups Do Prospective PSPNET Clients Belong to?

**Figure 1:**
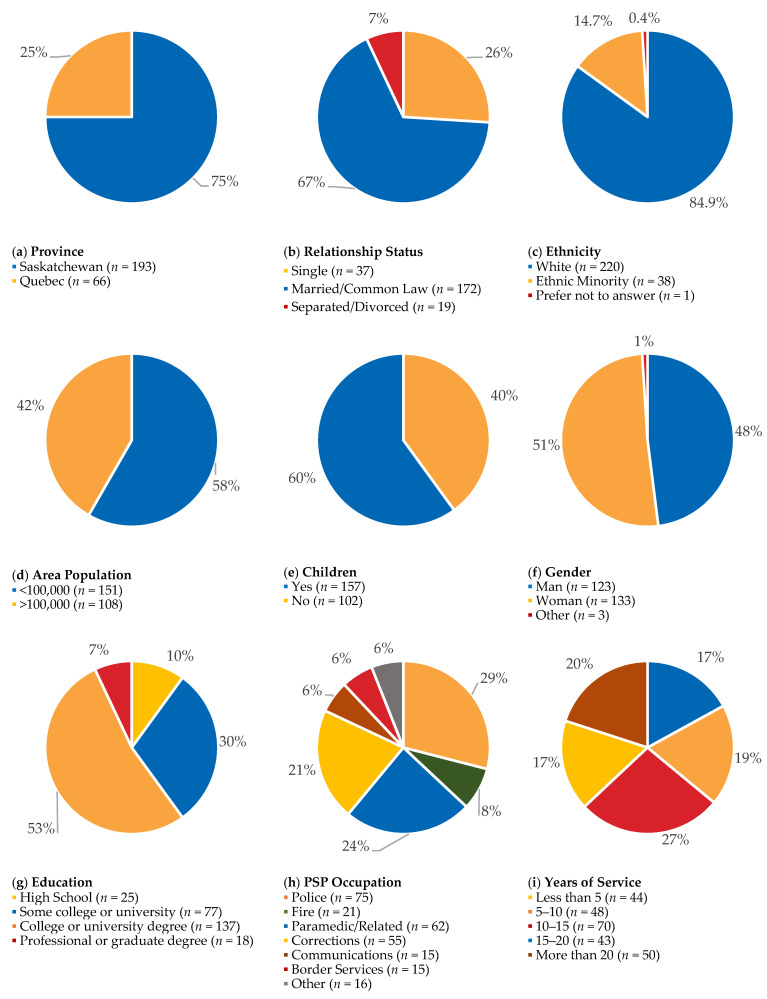
Participant demographic characteristics.

Participants (*N* = 259) were between 19 and 65 years of age (*M* = 40.19, *SD* = 9.82). Most participants were from Saskatchewan (74.5%), self-identified as white (84.9%), and self-identified as women (51.4%). Most participants reported having completed some college or university (29.7%) or having a college or undergraduate university degree (29%). Most participants reported being married or common-law (66.4%) and having children (60.6%). Participants came from diverse vocations, with most reporting being police (28.6%), paramedics (23.2%), or correctional workers (20.8%). Additional details on participant demographic characteristics are presented in Figure 1.

### 3.2. How Did Prospective Clients First Hear about PSPNET?

**Figure 2:**
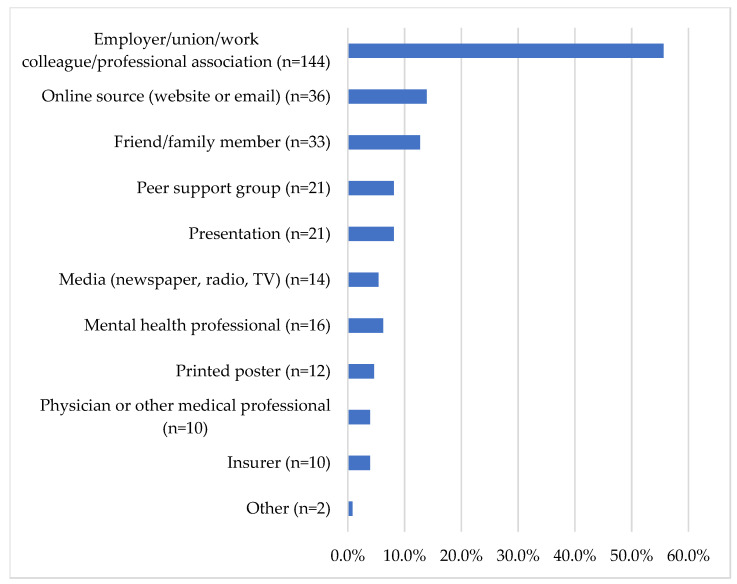
How did prospective clients first hear about PSPNET?

PSP reported learning about PSPNET and the associated service through several different mediums. Most prospective PSPNET clients reported having initially heard about PSPNET through an employer, union, work colleague, or professional association (55.6%). Prospective clients also reported learning about PSPNET through online sources (13.9%), such as websites or email, and from friends or family members (12.7%). Additional details on the mediums reported are presented in Figure 2.

### 3.3. How Do Prospective Clients Perceive ICBT?

Prospective clients reported positive beliefs about ICBT, with an average expected improvement in symptoms of 55% (*SD* = 22.64). Results from the CEQ showed that most prospective clients reported believing ICBT would be “somewhat effective” to “very effective” in improving their functioning (*M* = 6.28, *SD* = 1.7), that they would be “somewhat confident” to “very confident” in recommending ICBT to a friend (*M* = 6.3, *SD* = 1.9), and that ICBT seems “somewhat logical” to “very logical” (*M* = 6.94, *SD* = 1.76).

### 3.4. What Motivations Do Prospective Clients Report for Seeking ICBT?

**Figure 3:**
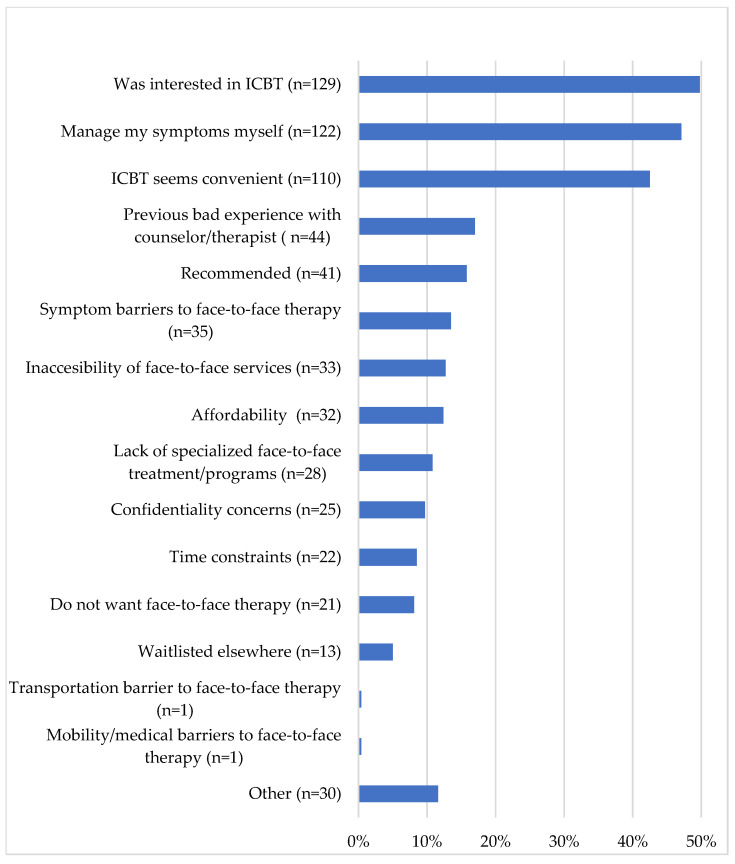
Reasons for seeking ICBT.

PSP reported several reasons for seeking ICBT, and most of the sample (74.1%) reported multiple reasons. The most commonly reported reasons for seeking ICBT included having heard about ICBT and wanting to try it (49.8%), wanting to learn to manage symptoms independently (47.1%), and the perceived convenience of ICBT (42.5%). Other common reasons for seeking ICBT included not feeling understood by a previous counsellor or therapist (17%), having had ICBT recommended to them (15.8%), and having difficulty attending face-to-face therapy as a consequence of their mental health (13.5%). Participants who selected more reasons for their interest in ICBT were more likely to enrol in the course (*t*(237) = −2.781, *p* = 0.006). The frequency of these reasons and several additional reasons for seeking ICBT are presented in Figure 3.

We also conducted a thematic analysis of prospective clients’ stated motivations for seeking ICBT. The most commonly reported motivation for seeking ICBT was dealing with perceived symptoms, which was reported by 126 (52%) prospective clients. All themes identified in the comments of three or more clients are displayed in Table 2 below.

### 3.5. What Past and Current Mental Health Treatments Do PSP Report Having Tried?

**Figure 4:**
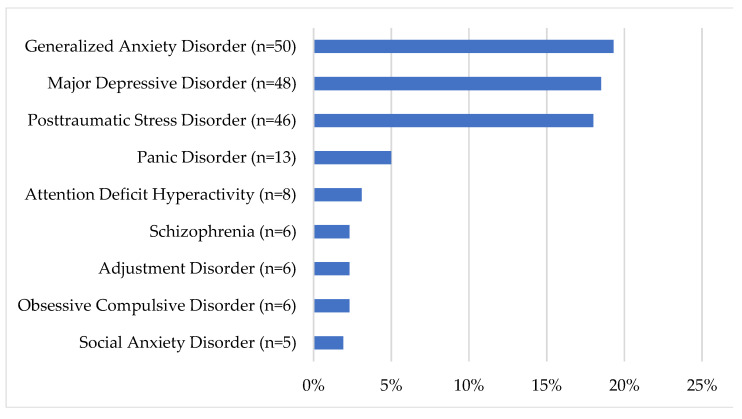
Prior diagnoses of mental disorders.

**Figure 5:**
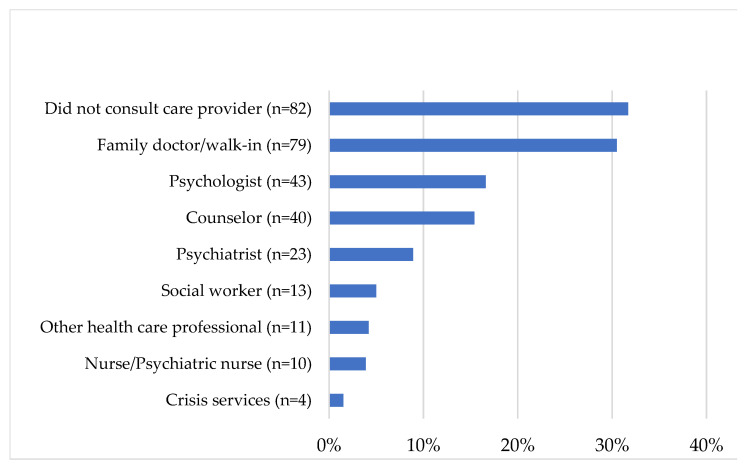
Prior support-seeking.

Most prospective PSPNET clients (52.5%) reported having previously consulted a healthcare or mental healthcare professional about mental health issues, although many (31.7%) did not (15.8% did not respond). Family doctors and walk-in clinics were the most commonly sought sources of care (30.5%), followed by psychologists (16.6%) and counselors (15.4%). Some PSP (27.8%) also reported having previously attended a self-help group, alcohol or drug treatment program, or an occupational stress injury program. Approximately half of our sample (44.8%) reported a prior diagnosis of a mental disorder (see Figure 4 for details). Many reported having previously been required to take time off work due to their mental health (*n* = 126, 48.6%) or having taken medication for mental health reasons (*n* = 96, 37.1%). A minority of clients also reported having received disability benefits (*n* = 19, 7.3%) or having been admitted to a health care institution (*n* = 4, 1.5%). Additional details on prior support seeking are presented in Figure 5.

### 3.6. What Are the Current Clinical Characteristics of Prospective PSPNET Clients?

**Figure 6:**
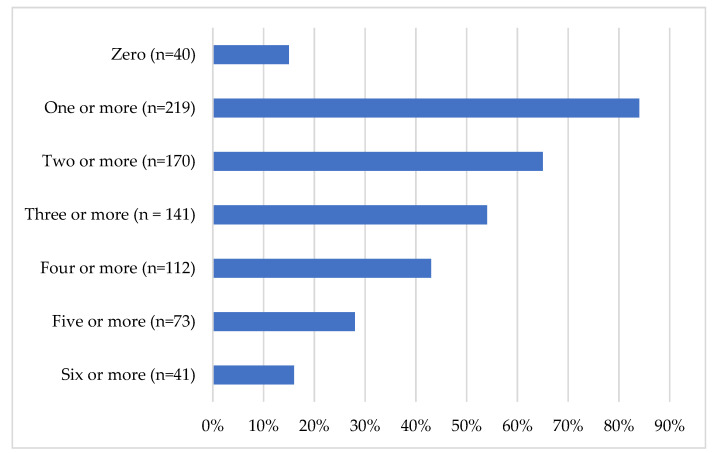
Number of clinically significant symptom areas.

In completing the PHQ-9, GAD-7, PCL-5, PDSS-SR, SIAS-6/SPS-6 or SIPS, DAR-5, AUDIT, and DUDIT, most prospective PSPNET clients screened positive for at least one mental disorder (84.7%), and two in three (65.8%) screened positive for two or more, but some reported symptoms below the clinical range on all measures (15.4%). The number of measures on which clients scored above clinical cut-offs are shown in Figure 6 below.

**Figure 7:**
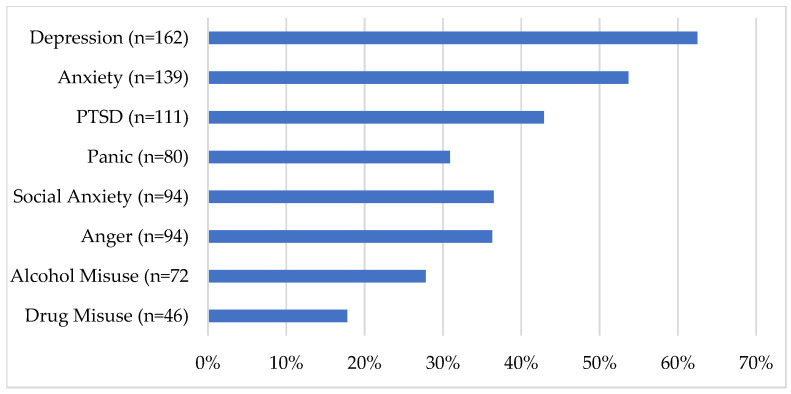
Percentage of PSP reporting clinically significant symptoms on each measure.

Prospective clients screened positive on measures of many mental health problems, including measures of MDD (PHQ-9), GAD (GAD-7), PTSD (PCL-5), panic disorder (PDSS-SR), social anxiety disorder (SIAS-6/SPS-6 or SIPS), anger (DAR-5), alcohol use disorder (AUDIT), and drug use disorder (DUDIT). The percentage of PSP who screened positive for each of these disorders is shown in Figure 7 below. Enrollers and nonenrollers did not vary statistically significantly in their symptom measure scores or their clinical status. Men had higher total scores on the AUDIT than did women (*t*(221) = −3.515, *p* < 0.001). No other gender differences were identified.

## 4. Discussion

### 4.1. Principal Findings

PSP in Canada and other countries experience high rates of mental health problems and face barriers to treatment. Preliminary evidence suggests that ICBT is an acceptable, accessible, and effective treatment for PSP populations, but little is known about why PSP seek ICBT. The current results provide important insights regarding the treatment-seeking experiences of PSP who seek ICBT.

Men and women were approximately equally represented in the current sample. Most Canadian PSP are men, but past research suggests that PSP women experience higher rates of mental health disorders than PSP men [4], and women report more favorable attitudes toward ICBT [27]; as such, the relatively even representation of men and women was unsurprising. The underrepresentation of PSP from Quebec is due, at least in part, to the fact that PSPNET first began offering services in Saskatchewan. Police, paramedics, and correctional workers accounted for nearly three quarters of the sample. There were fewer PSP from other occupational groups, possibly because some other groups are smaller (e.g., public safety communicators) or experience relatively lower rates of mental health problems (e.g., firefighters [4]); however, differences in uptake across PSP occupational groups could also be due to differences in how PSP organizations have helped inform their members about PSPNET’s services.

Indeed, most participants in the current sample (56%) reported having learned about PSPNET from employers, colleagues, unions, or professional associations, suggesting that engaging with PSP organizations has been a useful avenue for facilitating uptake of ICBT in this population. Prospective clients’ perceptions of ICBT, as indicated by their responses to the CEQ, were relatively positive and closely paralleled perceptions of a general PSP sample in a previous study [27]. Further efforts to reduce mental health stigma among PSP (e.g., the Road to Mental Readiness program [15]) may help further improve perceptions of ICBT and other treatments in the future.

PSP reported diverse motivations for seeking ICBT. Overcoming mental health problems was one of the most commonly cited motivations in both the quantitative and qualitative results. Other common motivations included the perceived convenience of ICBT and a desire among PSP to learn coping skills to manage symptoms by themselves. These motivations are consistent with the advantages of ICBT that PSP have described in past research [12,27]. The motivation for PSP to learn to manage their symptoms independently may be related to the stigma PSP have been found to experience for seeking mental healthcare [12,14]. The quantitative results showed that many clients reported facing barriers to face-to-face therapy (e.g., unaffordability of treatment, time constraints, concerns about mental health stigma). Interestingly, some PSP reported having had a bad experience with a counselor or therapist in the past, which may suggest a need for specialized services tailored for PSP. The qualitative results also showed that some PSP were motivated by a desire to improve their family functioning, learn skills to help coworkers, or pursue self-betterment, among other motives.

Most prospective PSPNET clients reported clinically significant symptoms of at least one mental disorder, and over half the sample reported clinically significant symptoms of three or more. Consistent with prior research [4], the most common disorders for which PSP reported clinically significant symptoms were MDD, GAD, and PTSD. Relatively few PSP (31.7%) reported not having previously consulted a healthcare or mental healthcare provider about mental health challenges, suggesting that existing mental healthcare options may have been unsuccessful or only partially successful for many of the PSP in the current sample. This could also indicate a need for ongoing services due to repeated trauma experienced by PSP [3].

### 4.2. Key Implications

Of the many results reported in this paper, there are three that we believe have particularly important implications for researchers and others involved in the development, delivery, evaluation, and funding of mental healthcare services for PSP. First, our results highlight the usefulness of partnering with PSP organizations to facilitate successful implementation of tailored mental health services for PSP. Second, many prospective clients reported being motivated to learn to manage their own symptoms of mental health problems, suggesting that skills-based and resilience-building treatment approaches may be a particularly good fit for this population. Third, most of our sample reported elevated symptoms of multiple mental disorders despite prior treatments, suggesting that PSP are a clinically complex population that may require transdiagnostic, specialized, and/or ongoing mental health supports.

### 4.3. Limitations and Future Directions

The current study has several limitations that highlight opportunities for future research. First, the current study was not designed to evaluate the ICBT services provided by PSPNET; however, the initial outcomes have previously been published [30]. Second, the current study explores only the experiences of PSP who signed up for PSPNET and provides no information on PSP who did not sign up for PSPNET. Third, the generalizability of the current results to PSP outside of Saskatchewan and Quebec remains unclear. Fourth, the current study was conducted during the COVID-19 pandemic, and the extent to which our results were influenced by the pandemic remains unclear. We have described the impacts of the pandemic on prospective PSPNET clients in a separate study, finding that they reported facing novel emotional challenges (e.g., social isolation, boredom, and fear) and logistical challenges (e.g., related to childcare, finances, and work) but generally did not report being severely impacted [56]. Fifth, certain results (e.g., concerning how PSP heard about PSPNET or which PSP occupations were most represented) were influenced by PSPNET’s unique outreach strategies and may not generalize well to other ICBT providers. Finally, researchers could explore how other technologies (e.g., digital mental health interventions that do not use a cognitive behavioural therapy approach, artificial intelligence interventions [57], and interventions carried out by social robots [58]) could help address the high rates of mental health problems among PSP in the future.

## 5. Conclusions

PSP report high rates of mental health problems and numerous barriers to mental healthcare. Preliminary results show that tailored ICBT is effective for treating symptoms of various mental disorders among PSP, but relatively little is known about the experiences of PSP who seek tailored ICBT. The current study provides insights into the treatment-seeking experiences of PSP interested in ICBT and has several important implications: partnering with PSP organizations appears to be a useful avenue for outreach to PSP; PSP appear to be motivated to learn skills to self-manage mental health challenges; and PSP appear to be a clinically complex population that may require specialized, transdiagnostic, and/or ongoing mental health supports.

## Figures and Tables

**Table 1:** Self-report measures.

Measure	Construct Measured	Number of Items	Item Score Range	Clinical Significance Cut-Off Score	Cronbach’s Alpha
PHQ-9 [38]	MDD	9	0–3	10	0.87
GAD-7 [39]	GAD	7	0–3	10	0.89
PCL-5 [40]	PTSD	20	0–4	33	0.95
PDSS-SR [41]	Panic Disorder	7	0–4	8	0.92
SIAS-6/SPS-6 [42]	Social Anxiety	12	0–4	7 and 2 ^1^	0.93
SIPS (French) [43,44,45]	Social Anxiety	14	0–4	12	0.94
DAR-5 [46]	Anger	5	1–5	12	0.85
SDS [49]	Disability	3	0–10	n/a ^2^	0.87
AUDIT [47]	Alcohol Use	10	0–4	6/8 ^3^	0.85
DUDIT [48]	Drug Use	11	0–4	2/6 ^3^	0.89
CEQ [50]	Treatment Credibility	4	1–9, 0–100	n/a ^2^	n/a ^4^
Adapted Tic-P [51]	Health Service Use	18–83 ^5^	Not a scored measure	n/a ^2^	n/a ^4^

^1^ A positive screen on the SIAS-6/SPS-6 requires a score of 7 or greater on the SIAS-6 and a score of 2 or greater on the SPS-6 [42]. ^2^ The SDS, CEQ, and Adapted Tic-P do not have cut-off scores indicating clinical significance. ^3^ The cut-off score on the AUDIT is 8 or greater for men and 6 or greater for women. The cut-off score on the DUDIT is 6 or greater for men and 2 or greater for women [48]. ^4^ We could not calculate a Cronbach’s alpha for the CEQ because different items uses different responses options, and we could not calculate Cronbach’s alpha for the Adapted Tic-P because it is not a scored measure. ^5^ The number of items in the Adapted Tic-P varies due to item display logic.

**Table 2:** Results of thematic analysis of motivations for seeking ICBT.

Description of Theme	Example Quotes	Frequency of Theme, *n* (%)
Dealing with perceived symptoms	“Learning how to manage anxiety.”	126 (52)
Multiple reasons	“Improve my quality of life and self-view. Be better in my relationship and increase intimacy. Increase ability to focus and process.”	18 (7)
Desire to improve wellbeing	“Improve my life and mental health.”	16 (7)
Coping tools and stress management	“Adding additional coping skills and resources to my skill set.”	14 (6)
Convenience	“Ease of treatment working around shift work.”	10 (4)
Increasing knowledge to help myself	“Wanting to learn”; “Better myself.”	9 (4)
Taking course for family and relationships	“To be healthy for my children.”	7(3)
Complementing existing treatment	“My psychologist felt it would be appropriate and would assist or compliment the treatment outline he sees for me.”	6 (2)
Curious about ICBT	“I am open to try new things.”	6 (2)
Benefit others	“I’m trying the material out to provide support to other members of my [profession].”	4 (2)
Alternative to face-to-face therapy	“I would like help fixing my issues without having to go to regimented face to face therapy.”	3 (1)
Using ICBT to diagnose perceived symptoms	“To see if I have any issues or if what I am feeling is just normal.”	3 (1)
Other	“Simplicity.”	10 (4)

## Data Availability

Due to concerns regarding participant confidentiality, the data used in this study will not be made available.
